# Management strategies to mitigate burnout and turnover intention while enhancing patient safety in neonatal intensive care: an integrative review

**DOI:** 10.3389/fpubh.2026.1835328

**Published:** 2026-05-25

**Authors:** Xi Huang, Yingxin Li, Yanling Hu, Yuan Li, Qiong Chen

**Affiliations:** 1 Department of Neonatology Nursing, West China Second University Hospital, Sichuan University, Chengdu, China; 2 Key Laboratory of Birth Defects and Related Diseases of Women and Children, Ministry of Education, Sichuan University, Chengdu, China

**Keywords:** health workforce sustainability, job demands-resources model, neonatal intensive care unit, nurse burnout, occupational health, patient safety culture, public health management, turnover intention

## Abstract

**Background:**

Globally, the nursing workforce shortage in Neonatal Intensive Care Units (NICUs) is evolving into a critical public health challenge. NICU nurses face a dual burden of high technical demands and intensive emotional labor, making them a high-risk group for professional burnout. The occupational wellbeing of nurses not only impacts individual health but also directly correlates with the clinical safety outcomes of preterm and ill neonates through the “health impairment process.”

**Objective:**

This study aims to introduce the Job Demands-Resources (JD-R) model as a systemic management diagnostic framework to comprehensively evaluate organizational management interventions targeting burnout, turnover intention, and patient safety among NICU nurses. The ultimate goal is to identify public health pathways that safeguard healthcare workforce sustainability and enhance patient safety.

**Methods:**

Adopting Whittemore and Knafl's integrative review framework and strictly following the PRISMA 2020 guidelines, we systematically searched PubMed, CINAHL, Web of Science, and Embase for original studies published between 2015 and 2025. The Joanna Briggs Institute (JBI) checklists were utilized for quality appraisal. The JD-R model was applied for deductive categorization and narrative synthesis of the interventions.

**Results:**

Fourteen original studies were included, 11 of which provided high-quality evidence. The findings revealed: (1) Optimization of job demands: Implementing flexible scheduling and Developmental Care Partners (DCP) reduced cognitive and physical loads, significantly mitigating burnout. (2) Enhancement of job resources: Mentoring programs, institutionalized career advancement pathways (e.g., ENNP roles), and participatory management strategies (e.g., “World Café” workshops) were identified as core drivers for increasing retention intentions. (3) Public health outcome linkage: Nurse burnout was positively correlated with healthcare-associated infection (HAI) rates. Strengthening nurse resilience through organizational resource injection proved to be an effective strategy for improving safety outcomes in vulnerable infant populations.

**Conclusions:**

NICU nursing management must transition from “individual crisis response” to “public health system governance.” Resource building based on the JD-R model, including institutionalized career paths and psychological resilience support, functions not merely as a management tool but as a critical defensive strategy for the sustainable development of the healthcare workforce and the safety of newborns.

## 1 Introduction

The sustainability of the global nursing workforce is confronting critical challenges. This is not merely an internal management dilemma within hospitals, but a pivotal public health issue that fundamentally impacts the stability and equity of healthcare systems worldwide. According to the latest reports from the World Health Organization (WHO) and the International Council of Nurses (ICN), although the size of the nursing workforce has grown, it is estimated that the world will still face a shortage of millions of nurses by 2025. This persistent shortage significantly threatens the stability and safety of global healthcare systems (1, 2). Against this macro background, the Neonatal Intensive Care Unit (NICU), as a highly specialized medical environment, faces unique pressures. Unlike general adult wards, NICU nurses must not only master complex life-support technologies to care for very low birth weight infants (VLBW) but also undertake high-intensity “emotional labor,” providing family-centered care and support to parents in states of extreme anxiety while treating their critically ill infants (3, 4). This dual burden of high technical requirements and intense emotional involvement poses a severe challenge to traditional nursing support systems, making NICU nurses a high-risk group for professional burnout, with stress levels often significantly higher than those in general nursing fields (5). To protect the health rights of VLBW infants, we must first protect the nurses who care for them. Given the extreme emotional and technical stress in the NICU, supporting nurse wellbeing is no longer just a management choice, it is a vital public health strategy to ensure the safety of our most vulnerable newborns.

According to the Job Demands-Resources (JD-R) model, when job demands (such as workload overload and high emotional demands) persistently exceed job resources (such as social support and job autonomy), a “health impairment process” is triggered, leading to severe professional burnout (6, 7). In the NICU environment, burnout is not only an individual occupational crisis but also a direct threat to patient safety. Empirical studies have shown that professional burnout can weaken nurses' cognitive control, leading to distractions and decision-making errors, thereby significantly increasing the risk of healthcare-associated infections (HAIs) (e.g., catheter-related bloodstream infections) and medication errors (8, 9). Furthermore, burnout is one of the strongest predictors of turnover intention. The loss of experienced NICU nurses not only causes a disruption in the continuity of care but also exacerbates poor patient safety outcomes by increasing the workload of the remaining nurses, creating a vicious cycle of “burnout-turnover-declining safety levels” (10, 11).

Although extensive research has investigated the relationship between nurse burnout and patient safety, for instance, a recent systematic review by Li et al. (12) confirmed the negative impact of nurse burnout on patient safety, satisfaction, and quality of care, limitations persist in the current synthesis of evidence. First, most existing reviews focus on adult ICUs or general nursing populations, failing to fully consider the specific needs for intervention strategies arising from the unique emotional labor characteristics of the NICU. Second, existing literature focuses largely on prevalence or correlation analyses, lacking a systematic integration of “management interventions” based on the JD-R framework. This “evidence-practice gap” makes it difficult for managers to scientifically prioritize interventions in the face of resource constraints. Therefore, nursing managers urgently require evidence-based knowledge on which specific organizational, leadership, or individual interventions, such as Magnet Hospital recognition (13) or mindfulness training (14), can effectively break this vicious cycle.

Hence, this integrative review is designed to systematically analyze and assess management strategies for NICU nursing burnout, retention, and safety outcomes. It leverages the JD-R model as an innovative “management diagnostic framework” to uncover how effective interventions catalyze improvements in nurse welfare and infant health through strategic resource allocation. The findings offer critical evidence-based insights for strengthening the resilience and sustainability of the global health workforce.

## 2 Methods

### 2.1 Design

This review was conducted using the integrative review methodological framework proposed by Whittemore and Knafl (15). This method encompasses five stages: problem identification, literature search, data evaluation, data analysis, and presentation of results. The unique advantage of this methodology lies in its ability to incorporate both experimental and non-experimental research, allowing for a comprehensive, multi-dimensional understanding of the complex interactions between burnout, turnover intention, and patient safety among NICU nurses. Furthermore, the reporting process of this review strictly followed the PRISMA 2020 (Preferred Reporting Items for Systematic Reviews and Meta-Analyses) guidelines (16) to ensure transparency, reproducibility, and methodological rigor.

### 2.2 Protocol and registration

In accordance with the PRISMA 2020 guidelines (Item 24a), it is noted that this integrative review was not formally registered in a public registry such as PROSPERO. The decision was based on the specific methodological nature of the integrative review framework by Whittemore and Knafl (15), which incorporates a diverse range of empirical and theoretical evidence that has historically fallen outside the primary scope of standard systematic review registries. The authors acknowledge that the absence of prior registration may increase the risk of *post-hoc* methodological decisions. To mitigate this risk and ensure transparency, the research process strictly adhered to a predefined PEO framework, the five-stage analysis process proposed by Whittemore and Knafl (15), and the reporting standards of the PRISMA 2020 statement.

### 2.3 Search strategy

A systematic search was performed across four authoritative electronic databases: PubMed, CINAHL (via EBSCO), Web of Science, and Embase. The search period spanned from January 1, 2015, to November 2025, aimed at encompassing the latest evidence from the COVID-19 pandemic and the post-pandemic era. The search strategy was constructed based on the PEO (Population, Exposure, Outcome) framework to ensure maximum sensitivity. The PEO framework was selected in preference to the traditional PICOS (Population, Intervention, Comparison, Outcome, Study design) framework for two primary reasons. First, “professional burnout” in the NICU context is predominantly examined as an “Exposure” or an existing condition in observational literature, rather than a strictly assigned “Intervention,” making the PEO structure more scientifically precise for this topic. Second, the PEO framework is highly congruent with the Whittemore and Knafl (15) integrative review methodology, which supports the synthesis of diverse evidence, including experimental, quasi-experimental, and qualitative research, thereby providing a more holistic diagnostic view of nursing management issues. During the search process, terms for “Population” (NICU nurses) and “Exposure” (burnout/turnover) were logically combined. Notably, terms related to “Patient Safety” were not used as search limiters; instead, they served as strict inclusion criteria during the title/abstract and full-text screening stages. This strategy was adopted to avoid overlooking studies that regarded patient safety as a secondary outcome or did not explicitly index it in the title/abstract. A combination of Medical Subject Headings (MeSH) and free-text keywords was employed. To ensure the relevance and precision of the results, all keyword searches were restricted to the title and abstract fields. The search process utilized Boolean operators (AND, OR) to logically connect the following three core concept groups:

Population: “Intensive Care Units, Neonatal” OR “NICU” OR “neonatal nurs^*^” OR “neonatal intensive care nurs^*^”.

Exposure/Intervention: “Burnout, Professional” OR “emotional exhaustion” OR “depersonalization” OR “turnover intention” OR “personnel turnover”.

Outcome: “Patient Safety” OR “medical errors” OR “hospital-acquired infection” OR “CLABSI” OR “unplanned extubation”.

The complete search strategy using PubMed as an example is detailed in Table 1. Search strategies for the other databases (CINAHL, Web of Science, and Embase) were appropriately adjusted based on their specific thesauri and syntax (see Supplementary Table S15 in the Supplementary materials). Furthermore, the search was restricted to peer-reviewed journal articles published in English. To identify any relevant literature potentially missed by the electronic database search, a manual “snowball” search was conducted on the reference lists of all included studies; no additional studies meeting the inclusion criteria were discovered through this process.

**Table 1:** PubMed database search strategy.

Step	Search Query/Terms	Results
#1	Population: (“Intensive Care Units, Neonatal”[Mesh] OR “Neonatal Nursing”[Mesh] OR “Nurses, Neonatal”[Mesh] OR “NICU”[Title/Abstract] OR “neonatal intensive care”[Title/Abstract] OR “neonatal nurs^*^”[Title/Abstract] OR “neonatal intensive care nurs^*^”[Title/Abstract])	47,795
#2	Exposure/Intervention Target: (“Burnout, Professional”[Mesh] OR “Burnout, Psychological”[Mesh] OR “Personnel Turnover”[Mesh] OR “Compassion Fatigue”[Mesh] OR “Empathy”[Mesh] OR “burnout”[Title/Abstract] OR “emotional exhaustion”[Title/Abstract] OR “depersonalization”[Title/Abstract] OR “turnover intention”[Title/Abstract] OR “intention to leave”[Title/Abstract] OR “personnel turnover”[Title/Abstract] OR “compassion fatigue”[Title/Abstract] OR “secondary traumatic stress”[Title/Abstract] OR “resilience”[Title/Abstract] OR “retention”[Title/Abstract])	393,271
#3	Combination: #1 AND #2	570
#4	Filters: Limit to English language, Publication date from 2015/01/01 to 2025/11/30	368^*^

^*^To ensure maximum sensitivity, “Patient Safety” and related outcomes were applied as inclusion criteria during the manual screening process rather than as keyword limiters in the initial search strategy.

### 2.4 Inclusion and exclusion criteria

The literature screening criteria are consistent with the aforementioned search strategy and are established based on the PEO framework to accommodate the mixed-methods nature of the included evidence. This approach ensures that studies exploring the associations between the NICU work environment (Exposure) and clinical safety outcomes (Outcome) are captured alongside interventional management evaluations.

Inclusion Criteria:

(1) Study Type: Peer-reviewed original empirical research, including quantitative, qualitative, or mixed-methods designs.
(2) Population: Registered nurses (RNs) whose work setting is explicitly identified as the NICU. For studies involving mixed multidisciplinary samples (e.g., physicians, respiratory therapists, and nurses), inclusion was contingent upon: (a) the availability of independent NICU nurse subgroup data, or (b) the study's focus on a unit-wide management intervention where nurses constituted the primary portion of the sample (e.g., >50%) and the results reflected the collective nursing work environment.
(3) Research Content: Studies must meet at least one of the following conditions: (a) Empirical investigation of the association between occupational burnout/turnover intention and patient safety outcomes; (b) Evaluation of managerial interventions aimed at addressing the aforementioned issues.
(4) Language: Articles published exclusively in English.

Exclusion Criteria:

(1) Non-specific Population: Studies focusing solely on general pediatric or adult ICU nurses that lack a specific subgroup analysis for NICU nurses.
(2) Publication Type: Gray literature, conference abstracts, editorials, expert opinions, and policy reports. While these sources often contain valuable workforce policies, they were excluded to ensure that all synthesized evidence met the threshold for rigorous methodological quality appraisal using the JBI checklists.
(3) Lack of Data: Theoretical articles or literature reviews that lack original empirical data support.

### 2.5 Study selection and quality assessment

#### 2.5.1 Literature selection process

**Figure 1:**
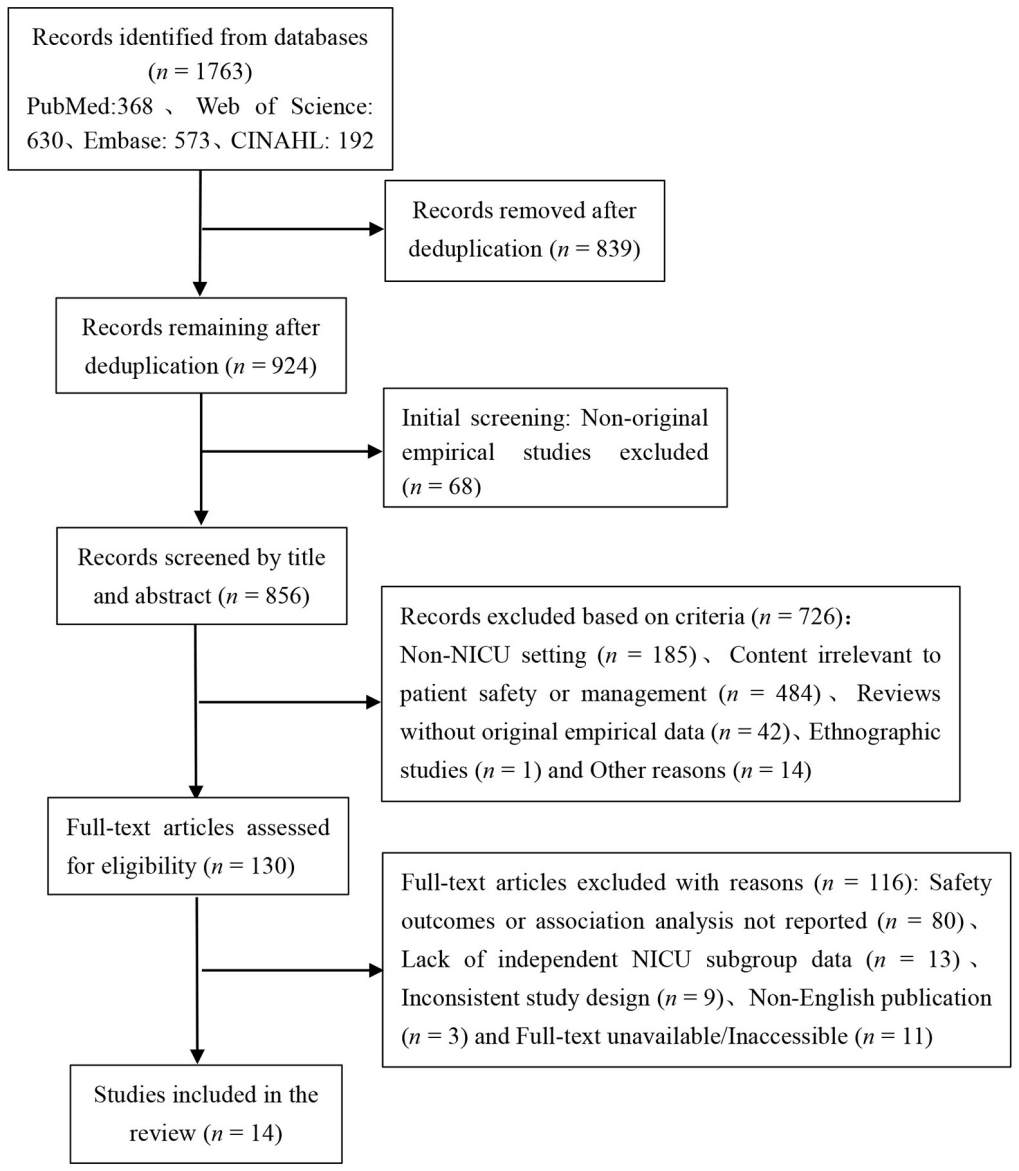
Flowchart of literature search and screening process.

Two researchers independently conducted the literature screening. The literature selection and reporting process strictly adhered to the PRISMA 2020 standards, as detailed in the PRISMA flow diagram (Figure 1). This diagram illustrates the transition from initial identification to final study inclusion.

Initially, a total of 1,763 records were identified through searches across four databases: PubMed (*n* = 368), Web of Science (*n* = 630), Embase (*n* = 573), and CINAHL (*n* = 192). All records were imported into Zotero reference management software for deduplication; after 839 duplicates were removed, 924 unique records remained for initial screening.

The screening process was conducted in two stages:

Initial Screening Stage: Based on a review of titles and abstracts, 68 non-empirical studies were first excluded (including 13 conference papers, 39 books, 2 patents, 1 thesis, 7 expert opinions/commentaries, and 6 others). Subsequently, the researchers evaluated the remaining 856 records and excluded 726 that did not meet the criteria. Primary reasons for exclusion included: non-NICU settings (*n* = 185), content irrelevant to patient safety or management interventions (*n* = 484), literature reviews lacking original data (*n* = 42), ethnographic studies (*n* = 1), and other reasons (*n* = 14).

Full-text Review Stage: A total of 130 articles proceeded to the full-text reading stage. Following a detailed review, 116 articles were excluded based on specific criteria: (1) failure to report safety outcome measures or lack of direct association analysis between burnout and safety (*n* = 80); (2) lack of independent NICU subgroup data (*n* = 13); (3) inconsistent study design (*n* = 9); (4) non-English publication (*n* = 3); and (5) inaccessible full text (*n* = 11).

Ultimately, 14 original empirical studies met all criteria and were included in this integrative review. Any discrepancies during the screening process were resolved through discussion with a third researcher.

#### 2.5.2 Quality appraisal

To ensure the reliability of the evidence, two researchers independently assessed the methodological quality of the 14 included studies using the JBI Critical Appraisal Checklists provided by the Joanna Briggs Institute (17). Specific tools were selected based on the research design of each study:

Quantitative Studies: Analytical cross-sectional studies (*n* = 6), cohort studies (*n* = 1), quasi-experimental studies (*n* = 2), and randomized controlled trials (*n* = 2).

Qualitative and Other Research: Qualitative studies (*n* = 2) and text and opinion/expert opinion (*n* = 1).

The quality assessment was quantified following the scoring logic proposed by Kmet et al. (18). The quality score was calculated using the following formula: Quality Score = (Number of “Yes” items / Total number of applicable items) × 100 %. Research quality was categorized into three levels (18): high quality (meeting ≥75% of criteria), moderate quality (50% – 74%), and low quality (< 50%). Any discrepancies in the appraisal were resolved through discussion or consultation with a third researcher. Studies with fatal methodological flaws, such as a lack of clear ethical approval or significant missing data reports, were excluded from the review.

The appraisal results indicated that the overall quality of the 14 included articles was excellent. Specifically, 11 studies were classified as high quality, 2 as moderate quality, and 1 as low quality. The low-quality study was retained because it provided a unique perspective on interventions, though its risk of bias was explicitly noted in the interpretation of the results. Detailed quality grading is presented in Table 2, and the specific appraisal items and rationales for each study are provided in the Supplementary materials (Supplementary Tables S1–S14). In accordance with the integrative review methodology, which synthesizes highly heterogeneous evidence (ranging from RCTs to qualitative narratives), a formal certainty assessment using structured frameworks such as GRADE or GRADE-CERQual was not performed. Instead, the strength of the evidence was derived through methodological quality appraisal using the JBI checklists, focusing on the internal validity and rigor of individual studies to guide the narrative synthesis.

**Table 2:** Summary of quality assessment results of included studies.

Study no.	First author (Year)	Study design	Type of JBI appraisal tool	Quality score	Quality level
S1	Sauerland et al. (19)	Cross-sectional study	Analytical cross-sectional	87.50%	High quality
S2	Noah and Potas (20)	Cohort study	Cohort studies	81.80%	High quality
S3	Clubbs et al. (21)	Quasi-experimental study	Quasi-experimental	44.40%	Low quality
S4	Tawfik et al. (9)	Cross-sectional study	Analytical cross-sectional	100.00%	High quality
S5	Liska et al. (22)	Randomized controlled trial (RCT)	Rct	69.20%	Moderate quality
S6	Moss (23)	Quasi-experimental study	Quasi-experimental	88.90%	High quality
S7	Tawfik et al. (24)	Cross-sectional study	Analytical cross-sectional	100.00%	High quality
S8	Jones and Ramsbottom (25)	Expert opinion	Textual evidence & opinion	100.00%	High quality
S9	Sano et al. (26)	Cross-sectional study	Analytical cross-sectional	100.00%	High quality
S10	Yu et al. (27)	Cross-sectional study	Analytical cross-sectional	100.00%	High quality
S11	Trajkovski et al. (28)	Qualitative study	Qualitative research	100.00%	High quality
S12	Asadollah et al. (29)	Randomized controlled trial (RCT)	Rct	76.92%	High quality
S13	Walden et al. (30)	Qualitative study	Qualitative research	70.00%	Moderate quality
S14	Deepak et al. (31)	Cross-sectional study	Analytical cross-sectional	100.00%	High quality

S1–S14 refer to the IDs of the included studies; for the detailed item-by-item quality appraisal and evidence for each study based on JBI checklists, please refer to Supplementary Tables S1–S14 in the Supplementary materials.

### 2.6 Data extraction

Data extraction was performed independently by two researchers using a pilot-tested standardized form. Any discrepancies arising during the process were resolved by re-examining the original literature and reaching a consensus through team discussion. The extracted data encompassed the following four dimensions (refer to Table 3: Summary of the basic characteristics of included studies):

**Table 3:** Summary of the basic characteristics of included studies.

	First author (Year)/Country	Level of evidence (LoE)	Study design/Sample size (*n*)	Measurement tools (Burnout/ Turnover/ Safety)	Intervention details (Duration/ Frequency)	Statistical analysis methods	Main results/Management intervention details
S5	Liska et al. (22)/United States (USA)	Level 1	Randomized controlled trial (RCT); *n* = 59 multidisciplinary healthcare workers (including NICU nurses, physicians, and support staff)	CD-RISC (Resilience); ProQOL (Compassion Satisfaction)	Self-paced mindfulness intervention delivered via a web portal; 6-week duration	Paired *t*-test; Robust bootstrapping; Permutation test	Mindfulness practice enhanced resilience across the multidisciplinary sample; synthesis prioritized outcomes reflecting nursing professional stress reduction within the mixed cohort.
S12	Asadollah et al. (29)/Iran	Level 1	Randomized controlled trial (RCT); *n* = 66 NICU nurses	MBI (Job Burnout; α = 0.9)	Loving-Kindness Meditation (LKM) audio intervention; 1-month duration, 3 sessions per week	Paired and independent *t*-tests; Analysis of Variance (ANOVA); Intention-to-treat (ITT) analysis	Loving-Kindness Meditation (LKM) significantly reduced burnout among NICU nurses and serves as an effective individual-level intervention.
S3	Clubbs et al. (21)/United States (USA)	Level 2	Quasi-experimental study (pre-post design); NICU nurses in a community hospital	MBI-HSS (Burnout); Pediatric infection data extracted from Electronic Health Records (EHR)	Pre- and post-implementation comparison of the Developmental Care Partners (DCP) program	Independent samples *t*-test; Fisher's exact test; Relative Risk (RR) as an effect measure	Following the implementation of the Developmental Care Partners (DCP) program, nurse burnout decreased significantly without increasing infant infection rates.
S6	Moss (23)/United States (USA)	Level 2	Quasi-experimental study; Neonatal Nurse Practitioners (NNPs) in a Level IV NICU	MNPJSS (Job Satisfaction; α = 0.96); Intent to stay	Mentoring program; 4 measurement time points	One-way repeated measures ANOVA; Spearman's rank correlation analysis	Mentoring significantly improved Job Satisfaction and Intent to Stay among NNPs, buffering against turnover risk.
S2	Noah and Potas (20)/Somaliland	Level 3	Cohort study; *n* = 45 nurses and *n* = 72 neonates	MBI (Burnout); NSS (Stress); Objective clinical infection indicators (e.g., bloodstream, respiratory tract)	5-month follow-up period	Random-effects logistic regression; Poisson regression; Panel data analysis	Nurse burnout was significantly associated with neonatal healthcare-associated infection (HAI) rates; high-stress environments directly increased the risk of infection.
S1	Sauerland et al. (19)/United States (USA)	Level 4	Analytical cross-sectional study; Registered Nurses (RNs) in the NICU/PICU of an academic medical center	HECS (Ethical Climate; α = 0.94); MDS-R (Moral Distress; α = 0.93)	N/A (Cross-sectional survey)	Pearson correlation analysis	Organizational ethical climate was significantly negatively correlated with nurse moral distress; a positive climate helps alleviate the psychological burden on nurses.
S4	Tawfik et al. (9)/United States (USA)	Level 4	Analytical cross-sectional study; *n* = 2,073 multidisciplinary providers (RNs, NPs, MDs, and RTs)	MBI (4-item Emotional Exhaustion subscale); HAI rates defined by CPQCC	N/A (Cross-sectional survey)	Multilevel logistic regression; Hierarchical modeling; adjusted for clinical risk factors and provider roles	Personnel's perception of “working too hard” was significantly associated with HAIs; nursing-specific impact was highlighted as nurses constitute the primary unit workforce influencing safety.
S7	Tawfik et al. (24)/United States (USA)	Level 4	Analytical cross-sectional study; *n* = 1,934 NICU workers (including physicians, nurses, and respiratory therapists)	MBI (Emotional Exhaustion; α = 0.85); Clinical data from CPQCC and OSHPD	N/A (Cross-sectional survey)	Multilevel logistic regression; Variable selection via LASSO models to account for multidisciplinary variance	Unit-level burnout prevalence was established; synthesis extracted organizational predictors that reflect the shared nursing work environment within the NICU.
S9	Sano et al. (26)/United States (USA)	Level 4	Analytical cross-sectional study; Registered Nurses (RNs) in Level III/IV NICUs in the Midwest	ProQOL (Compassion Fatigue); STSS (Traumatic Stress); OLBI (Burnout)	N/A (Cross-sectional survey)	Moderated mediation analysis; Structural Equation Modeling (SEM); 95% bootstrapping	Strong nurse-patient relationships and collaborative environments buffer against the negative consequences of providing care.
S10	Yu et al. (27)/South Korea	Level 4	Analytical cross-sectional study; NICU nurses in tertiary and general hospitals	AHRQ Hospital Survey on Patient Safety Culture; Nursing Quality of Care (NQoC); Turnover intention	N/A (Cross-sectional survey)	Hierarchical logistic regression analysis; G-Power sample size calculation	Perceptions of staffing levels, job satisfaction, and safety culture serve as core predictors of nurse-reported quality of care.
S14	Deepak et al. (31)/India	Level 4	Multicenter analytical cross-sectional study; healthcare providers from 8 tertiary hospitals	TAWS-16 (Occupational Stress); K10 (Psychological Distress)	N/A (Cross-sectional survey)	Multivariable logistic regression analysis; Chi-square (χ^2^) test; Spearman's correlation	Working hours, monthly mortality rates, and recent life events are key factors driving high levels of stress among NICU healthcare providers.
S8	Jones and Ramsbottom (25)/United Kingdom (UK)	Level 5	Textual evidence and expert opinion; NICU nursing population	Comprehensive assessment of recruitment, career development, and retention intention	Pathways for developing neonatal nurses to an enhanced level (ENNP)	Qualitative evidence synthesis (QES); Rigorous logical synthesis of the literature	Empowerment and Continuing Professional Development (CPD) are critical to improving nurse retention, with the ENNP role deepening the scope of clinical nursing care.
S11	Trajkovski et al. (28)/Australia	Qual	Qualitative study (participatory); senior nurses, scholars, and clinical researchers	Semi-structured discussions on recruitment, retention, and attrition (turnover)	World Café methodology workshop; 6 groups with rotating discussions	Braun & Clarke's inductive thematic analysis framework	Four strategic themes were generated to strengthen the neonatal nursing workforce, highlighting shared organizational responsibility.
S13	Walden et al. (30)/United States (USA)	Qual	Qualitative descriptive study; large-scale sample of NICU nurses	Subjective narratives of stressors (inability to sleep at night) and motivators (motivation to get up in the morning)	Online qualitative survey based on the Transactional Model of Stress and Coping (TMSC)	Thematic analysis	Core stressors, including workload, patient mortality, and interpersonal relationships, were identified, leading to organizational recommendations for resilience enhancement.

Level 1, Randomized Controlled Trial (RCT); Level 2, Quasi-experimental study; Level 3, Cohort study; Level 4, Cross-sectional study / Descriptive study; Level 5, Expert opinion / Textual evidence; Qual, Qualitative research.

Basic characteristics: Including first author, publication year, country/region, study design, sample size, and sampling method.

Measurement of key variables: Specific tools and measurement outcomes for occupational burnout (e.g., MBI-HSS), turnover intention (e.g., TIS-6), and patient safety indicators (e.g., nosocomial infection rates, medication errors).

Intervention details: For interventional studies, systematic extraction of the intervention type, duration, implementation frequency, and personnel involved, as well as the theoretical basis grounded in the Job Demands-Resources (JD-R) model.

JD-R theoretical categorization: Classification of factors into “Job Demands” (e.g., emotional labor, workload), “Job Resources” (e.g., social support, autonomy), and relevant organizational or patient outcome indicators based on the JD-R framework.

### 2.7 Data synthesis

**Figure 2:**
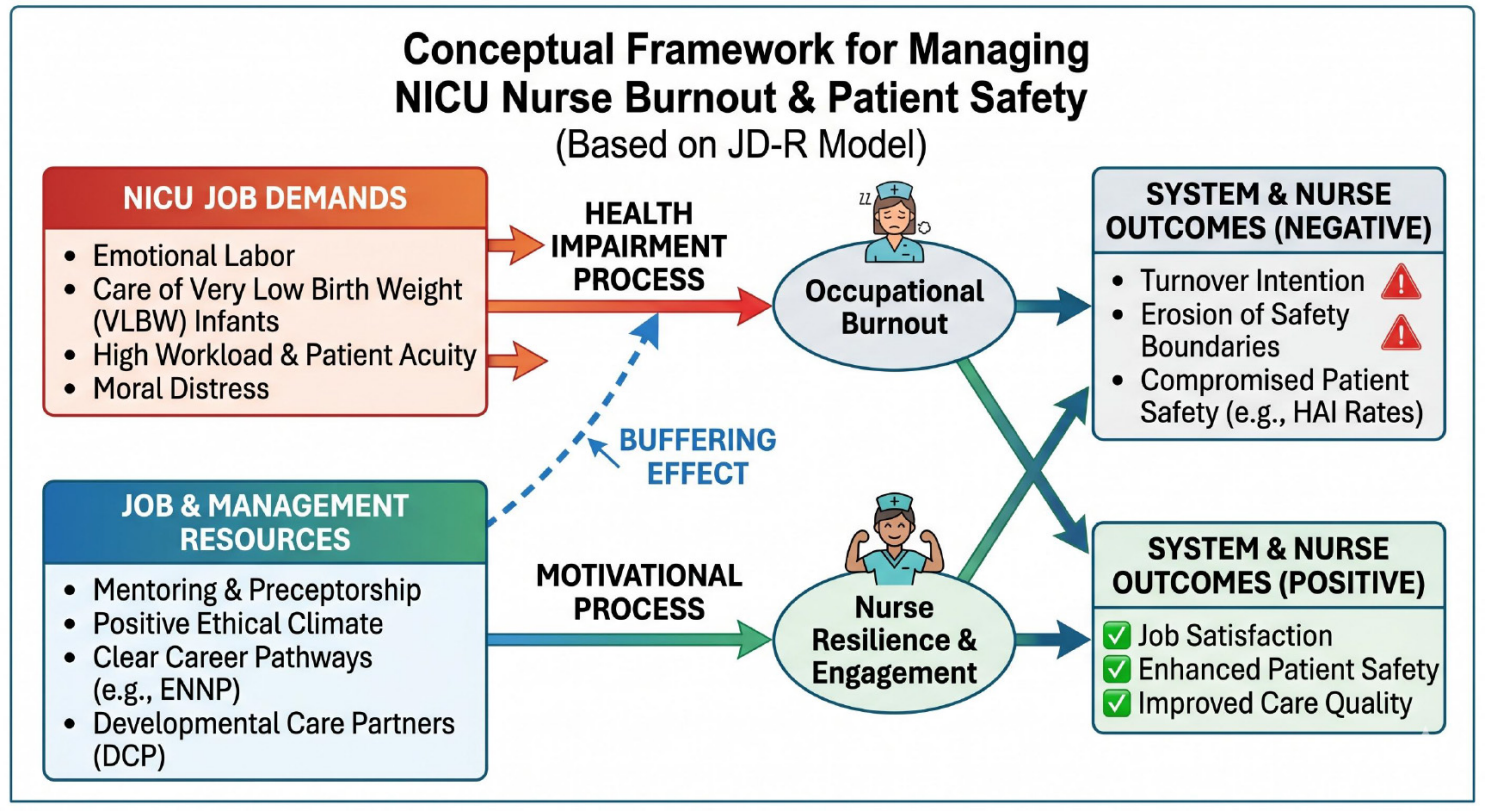
Conceptual framework for managing NICU nurse wellbeing and patient safety based on the JD-R model. This figure illustrates the dual pathways of the JD-R model tailored to the NICU context.

Due to significant clinical and methodological heterogeneity among the included studies regarding study design, interventions, and outcome measures (such as the use of diverse patient safety assessment tools), a quantitative meta-analysis was considered inappropriate. Therefore, this study followed the guidelines proposed by Popay et al. (32) and employed a narrative synthesis approach to analyze the data. The Job Demands-Resources (JD-R) model was utilized as the theoretical framework to perform a deductive categorization of the extracted data (illustrated in Figure 2). Based on the JD-R framework, the research findings were integrated into the following three major themes:

(1) Specific Job Demands in the NICU Environment: Identifying the key stressors that drive the “burnout-turnover-unsafe practice” vicious cycle.
(2) Impact of Nurse Burnout on Patient Safety Outcomes: Covering specific empirical associations with infection rates, medication errors, and near-miss events.
(3) Management Intervention Strategies Based on the JD-R Model: Categorizing interventions into organizational-level resource building, leadership support, and individual-level resource interventions.

- **Health Impairment Process:** Sustained high job demands (e.g., emotional labor, VLBW care) without adequate support trigger this process, leading to nurse burnout, increased turnover intention, and compromised patient safety (e.g., higher HAI rates).
- **Motivational Process:** The injection of organizational resources (e.g., mentoring, ENNP pathways) fosters nurse resilience and job satisfaction, ultimately enhancing the quality of neonatal care.
- **Buffering Effect:** Adequate job resources offset the negative impacts of high-intensity demands, serving as a critical defense line to break the “burnout-turnover-safety erosion” cycle.

Furthermore, to address studies utilizing multidisciplinary samples [e.g., Liska et al. (22), Tawfik et al. (24)], our synthesis prioritized the extraction of nursing-specific subgroup data. In cases where data were inextricably reported at the unit level (such as unit-wide mindfulness or management interventions), the findings were integrated into our synthesis under the premise that nurses constituted the majority of the healthcare workforce in these settings, and thus the outcomes predominantly reflected the collective nursing work environment.

### 2.8 Assessment of reporting bias

In accordance with PRISMA 2020 item 21, the risk of bias due to missing results (publication bias) was assessed narratively. Given the significant methodological heterogeneity and the descriptive nature of the synthesis, quantitative assessment tools such as funnel plots or Egger's test were considered inappropriate. Instead, potential reporting bias was evaluated by examining the geographical distribution of studies, the diversity of study designs, and the balance between positive and non-significant findings. The impact of limiting the search to peer-reviewed English-language journals was also critically appraised as a potential source of language and publication bias.

## 3 Results

### 3.1 Characteristics of included studies and quality assessment

A total of 14 original studies were included in this review, with publication dates spanning from 2015 to 2025. The studies demonstrate a broad geographical distribution, including the United States (*n* = 8) (9, 19, 21–24, 26, 30), and one study each from Somaliland (20), South Korea (27), the United Kingdom (25), Australia (28), Iran (29), and India (31). The research designs were diverse, comprising cross-sectional studies (*n* = 6), randomized controlled trials (*n* = 2), quasi-experimental studies (*n* = 2), qualitative studies (*n* = 2), a cohort study (*n* = 1), and textual evidence (*n* = 1). According to the methodological quality assessment, 11 studies were categorized as high quality, 2 as moderate quality, and 1 as low quality. Specifically, the quasi-experimental study by Clubbs et al. (21) was rated as low quality (44.4%) due to several methodological limitations, including the lack of a parallel control group, significant participant attrition (with only a 34% overlap between pre- and post-test groups), and lower internal consistency in certain burnout subscales. Despite these limitations, this study was retained as it provides the only empirical evidence within this review regarding the impact of a volunteer-led Developmental Care Partners (DCP) program on both nurse burnout and infant infection rates; its findings were interpreted with caution throughout the synthesis.

### 3.2 Specific job demands in the NICU: drivers of burnout

Guided by the JD-R theory, the high-intensity “job demands” faced by NICU nurses are the core factors leading to burnout.

(1) Clinical Workload and Emotional Stress: High workload, patient mortality, and complex interpersonal relationships were identified as the primary stressors for NICU nurses (30).
(2) Organizational and Environmental Factors: Prolonged working hours, high monthly mortality rates (31), and a poor ethical climate (e.g., moral distress) (19) significantly increased the psychological burden on nurses.
(3) Specific Skill Requirements: The high sensitivity and complex technical requirements associated with very low birth weight (VLBW) infants present a continuous dual challenge, both technical and emotional, to nursing staff (9).

When these job demands persist, they transform stress into occupational burnout through the “health impairment process,” serving as the catalyst for subsequent turnover and patient safety risks.

### 3.3 Impact of nurse burnout and turnover intention on patient safety outcomes

Empirical studies have confirmed a direct association between the professional wellbeing of NICU nurses and neonatal outcomes. However, it is important to acknowledge that the predominance of cross-sectional designs (43% of the included studies) in the current evidence base limits the ability to establish definitive causal directionality between these variables. Consequently, the findings summarized below should be interpreted primarily as significant correlations rather than established causal pathways.

(1) Increased Risk of Clinical Infections: Nurse burnout is significantly positively correlated with neonatal HAI rates (20). In high-pressure environments, evidence from multidisciplinary cohorts (including nurses, physicians, and respiratory therapists) indicates that emotional exhaustion among healthcare providers is strongly linked to HAIs in very low birth weight (VLBW) infants (9). Although these findings are derived from mixed-profession samples, they are integrated into this synthesis under the premise that nurses constitute the primary portion of the NICU workforce and their professional wellbeing is a fundamental driver of the unit's collective safety climate.
(2) Impairment of Perceived Quality of Care: Nurses' perceptions of staffing levels, job satisfaction, and safety culture scores serve as core predictors of their assessment of Nursing Quality of Care (NQoC) (27).
(3) Negative Ripple Effects of Turnover: Burnout is a primary driver of turnover intention. Research confirms (23) that the loss of experienced nurses not only disrupts the continuity of care but also is associated with deteriorated safety outcomes due to the increased workload imposed on the remaining staff, ultimately creating a vicious cycle of “burnout - turnover - declining safety.”

### 3.4 Management intervention strategies based on the JD-R framework

Research indicates that increasing “job resources” can effectively buffer the negative impacts of the aforementioned high job demands.

#### 3.4.1 Organizational-level resource building

(1) Mentoring and Career Advancement: Implementing mentoring programs significantly enhances job satisfaction among Neonatal Nurse Practitioners (NNPs) and buffers against turnover intention (23). Simultaneously, establishing pathways for nurses to achieve Enhanced Neonatal Nurse Practitioner (ENNP) status through continuing professional development is identified as a key strategy for improving retention (25).
(2) Participatory Workforce Development: Utilizing the “World Café” methodology to involve senior nurses in workforce development workshops fosters shared organizational responsibility and generates effective strategies for recruitment and retention (28).
(3) Optimizing Care Models: The implementation of the DCP program not only significantly reduces nurse burnout but has also been clinically proven not to increase the risk of infant infections (21).

#### 3.4.2 Leadership and cultural support

(1) Fostering an Ethical Climate: A positive organizational ethical climate helps alleviate moral distress among nurses and buffers the negative professional consequences associated with high-stakes care (19).
(2) Improving Collaborative Environments: Establishing strong nurse-patient relationships and a collaborative team environment can effectively mitigate compassion fatigue and burnout resulting from high-intensity nursing work (26).

#### 3.4.3 Individual-level resource regulation (resilience interventions)

(1) Mindfulness and Meditation Practices: Loving-Kindness Meditation (LKM) audio interventions have been clinically proven to significantly reduce job-related burnout among NICU nurses (29). Additionally, web-based mindfulness exercises have been shown to enhance resilience and compassion satisfaction (22). Notably, while the study by Liska et al. (22) involved a multidisciplinary sample of hospital-based healthcare workers, the positive psychological outcomes are highly applicable to the nursing workforce, which formed the core of the target unit.
(2) Stressor Identification and Coping: Identifying core stressors, such as workload and interpersonal relationships, assists organizations in formulating targeted recommendations to strengthen individual-level resilience (30).

In summary, by providing sufficient “job resources,” these management interventions effectively offset the health impairment caused by high-intensity NICU job demands, playing a critical buffering role.

## 4 Discussion

### 4.1 Systemic risks in the NICU environment: from burnout to safety erosion

The findings of this systematic review provide robust support for the “health impairment process” pathway within the Job Demands-Resources (JD-R) model (6, 7). The unique clinical environment of the NICU, particularly when caring for very low birth weight (VLBW) infants, is characterized by exceptionally high job demands (9, 24). In addition to mastering complex life-support technologies, nurses must perform high-intensity “emotional labor”, balancing the psychological support provided to anxious families with the acute management of fluctuations in neonatal conditions (3, 4). The results of this review indicate that when these demands chronically exceed available organizational resources, occupational burnout becomes an inevitable consequence (6, 7).

Crucially, burnout is not merely an individual professional crisis; it is strongly associated with clinical risk, presumably by impairing nurses' cognitive functions and attentional focus (8, 33). Nonetheless, because nearly half of the included studies rely on cross-sectional data, caution is required when assigning strict causality, as the temporal sequence, whether burnout precedes safety failures or vice versa, remains to be further validated through longitudinal research. Empirical evidence has confirmed a significant positive correlation between nurses' levels of emotional exhaustion and the rates of HAIs (9, 20, 24). Recent evidence further suggests that burnout diminishes nurses' sensitivity to latent safety hazards, creating a “bottleneck effect” in attentional monitoring. This cognitive strain increases the probability of medication errors or unplanned extubations (12, 34). In high-stakes environments like the NICU, where the margin for error is extremely narrow, the professional wellbeing of nurses serves as a vital defense line correlated with safeguarding neonatal lives (27, 31). From the lens of public health governance, nursing burnout must not be viewed as a sign of personal psychological fragility. Instead, it should be redefined as a critical early warning signal indicating a systemic disequilibrium of organizational resources.

### 4.2 The buffering effect of management resources: breaking the “burnout–turnover” Chain

According to the “motivational process” (or gain pathway) of the JD-R framework, effective management interventions can offset the negative impacts of high job demands by providing sufficient “job resources” (11, 13).

(1) Resource Building at the Organizational and Leadership Levels This review demonstrates that implementing mentoring programs is a core strategy for enhancing job satisfaction and buffering turnover intention (23). Guidance from senior nurses not only accelerates the clinical role adaptation of junior nurses but also significantly reduces practice anxiety stemming from technical inexperience (23). Furthermore, the introduction of innovative clinical processes, such as the DCP program, effectively mitigates nurse workload while optimizing infant developmental outcomes (21). A positive organizational ethical climate has also been proven to alleviate the moral distress nurses face during ethical conflicts, thereby preventing the accumulation of negative professional consequences (19).

(2) Resource Interventions at the Individual and Resilience Levels Beyond organizational support, enhancing nurses' personal psychological resources, particularly resilience, is equally vital. Mindfulness training and Loving-Kindness Meditation (LKM) serve as accessible psychological buffering tools that have been shown to significantly improve compassion satisfaction and alleviate traumatic stress (22, 29). Strengthening these internal resources empowers nurses to block the pathway from stress to occupational burnout, even when faced with high-intensity caregiving tasks (22, 29).

### 4.3 Retaining experienced nurses: public health value and economic implications

The findings of this systematic review further substantiate the central role of senior nurses in maintaining the organizational stability of the NICU (28). The loss of senior, highly experienced nursing personnel represents a severe depletion of the hospital's “professional human capital.” A systematic review by Bae (10) indicates that the cumulative sunk costs of losing a single registered nurse, encompassing recruitment, orientation, and initial productivity fluctuations, typically range from 0.25 to 3.0 times their annual salary. While a multiplier of 1.5 to 2.0 is often cited as a benchmark for critical care staff, this estimate is subject to significant uncertainty across different healthcare systems and economic contexts.

Notably, for highly specialized units like the NICU, these costs are likely to gravitate toward the higher end of the spectrum. This is primarily attributed to the intensive post-hire investment required, such as prolonged orientation periods (often exceeding 3–6 months) and complex technical training for neonatal life-support equipment, which significantly inflates replacement costs compared to general nursing units. From an organizational dynamics perspective, the unplanned departure of core staff members often triggers a “turnover contagion” effect. This phenomenon exacerbates the workload and psychological stress of the remaining personnel, ultimately eroding patient safety margins and representing a severe depletion of public health human capital (11).

Consequently, grounded in the “resource gain” logic of the JD-R framework, administrators should regard the refinement of professional development pathways, such as the “Enhanced Neonatal Nurse Practitioner” (ENNP) role, as a high-return-on-investment (ROI) safety governance strategy. By securing the retention of senior experts through institutionalized measures, they can effectively construct a robust safety defense system for the unit (25).

### 4.4 Practical implications for public health and nursing management

Grounded in the evidence-based findings of this review, this study proposes the following strategic recommendations for systemic governance:

(1) Transitioning from “Crisis Response” to “Systems Diagnosis”: Constructing a Dynamic Risk Monitoring Logic Using the JD-R Model.

Management should move beyond the traditional perception of burnout as an individual psychological issue and instead define it as a signal of systemic resource imbalance. It is recommended that the JD-R model be adopted as a management diagnostic tool (7) to dynamically monitor nurses' perceptions of staffing levels and their emotional exhaustion scores (24, 27), while establishing clinical safety thresholds. The essence of this management logic is the creation of an “early warning feedback loop.” When monitoring systems detect a significant spike in job demands (such as high monthly mortality rates or moral distress), “job resources” should be administratively injected (such as the temporary deployment of auxiliary support staff or providing psychological support) to prevent systemic safety accidents induced by resource depletion.

(2) Strengthening “Participatory Governance”: Enhancing Organizational Psychological Safety Through Empowerment.

The introduction of structured dialogue techniques, such as the “World Café” methodology (28), allows nurses across varying levels of seniority and hierarchy to engage in rotating discussions in a relaxed, informal setting. These sessions focus on core themes such as “optimizing ward governance” or “enhancing team retention.” The underlying managerial value of this approach lies in its ability to bolster nurses' structural empowerment and organizational identification through de-hierarchical communication. This “flattened governance” logic taps into the tacit wisdom of the nursing staff, transforming them from passive executors of policy into active co-decision-makers in ward governance. By granting professional autonomy to frontline nurses, organizations can effectively buffer the psychological stress associated with high-intensity work. This process cultivates an organizational culture rooted in “shared responsibility,” thereby reinforcing patient safety boundaries at a systems level.

(3) Digital Empowerment and Human Capital Protection

Administrators should examine scheduling and promotion systems through the lens of “human capital” appreciation. First, as an emerging and exploratory field, future research could consider further exploring AI-driven scheduling optimization algorithms (35) to ensure clinical safety while simultaneously addressing nurses' needs for perceived fairness and work-life balance, thereby mitigating the physical and emotional depletion caused by sub-optimal scheduling. Second, mentoring programs and formalized career progression pathways, such as the Enhanced Neonatal Nurse Practitioner (ENNP) role, should be recognized as high-Return on Investment (ROI) asset protection strategies. Given that the cumulative sunk costs associated with the loss of a single senior nurse can reach 1.5 to 2 times their annual salary (10), institutionalized career development serves as the optimal high-ROI management strategy for reducing exorbitant turnover costs and safeguarding the hospital's safety boundaries.

### 4.5 Limitations and future research directions

It should be acknowledged that the reliance on observational and cross-sectional studies reflects the inherent challenges in nursing management research. Randomized controlled trials (RCTs) are often limited by ethical constraints and practical feasibility when manipulating organizational or psychological variables in clinical settings. Consequently, this study employed an integrative review methodology, as proposed by Whittemore and Knafl (15), specifically to synthesize diverse research designs and provide a multi-dimensional understanding of the “burnout-turnover-safety” nexus within the complex NICU environment. Although this review provides multi-dimensional, evidence-based insights for NICU nursing management, several limitations remain due to the current state of available research:

Methodological Constraints: Regarding study design, nearly half of the 14 original studies included (*n* = 6) utilized a cross-sectional design. This “snapshot” approach to data collection makes it difficult to establish a definitive causal chain between occupational burnout, turnover intention, and patient safety over a longitudinal time series. Future research should prioritize longitudinal or prospective cohort studies to better capture the dynamic evolution of these variables.

Heterogeneity of Outcome Measures: There is significant heterogeneity in the measurement of outcome indicators. The definitions and assessment tools for “patient safety” were inconsistent across studies, encompassing objective clinical metrics such as HAI rates (e.g., S2, S4, S7) and subjective nurse-reported perceptions of Nursing Quality of Care (NQoC) (e.g., S10), while several studies did not directly measure safety outcomes (e.g., S1, S9, S11). This significant variability limits the ability to conduct robust cross-study comparisons and may introduce reporting bias, which should be considered when interpreting the synthesized evidence.

Publication, Language, and Geographical Bias: Regarding publication bias, this review is susceptible to several risks. First, the geographical distribution of the evidence is heavily concentrated in high-income countries (HICs), with the United States accounting for over 50% of the included studies. Second, the exclusion of gray literature and unpublished technical reports may have omitted non-significant findings or localized policy evidence that did not reach academic publication. Third, the “language barrier” (English-only restriction) likely introduces a systematic bias against LMIC-specific management interventions, potentially leading to an over-representation of Western-centric high-resource models (e.g., Magnet Hospital recognition). Although we systematically assessed the internal validity of individual studies using JBI checklists, the absence of a formal GRADE certainty assessment reflects the inherent challenges of integrating mixed-methods data. Consequently, the policy recommendations provided herein should be viewed as evidence-informed guidance with a moderate level of certainty, necessitating cautious implementation in diverse clinical contexts.

Sample Composition Overlap: While this review aimed to strictly focus on NICU nurses, a few included studies (particularly evaluating unit-wide management interventions or prevalence rates) utilized multidisciplinary samples encompassing physicians and respiratory therapists alongside nurses. Although nurses constituted the primary demographic in these studies, the inextricably mixed data may introduce slight confounding effects. However, this also reflects the authentic, interdisciplinary nature of NICU patient safety culture, where management interventions often target the collective team rather than isolated professions.

Future Directions for Research:

(1) Elevating Research Hierarchy and Longitudinal Depth: Future studies should prioritize high-quality Randomized Controlled Trials (RCTs) and prospective cohort studies to validate the long-term effectiveness of specific management interventions, such as mentoring and DCP programs. It is particularly crucial to move beyond inpatient safety indicators and explore the long-term impact of these interventions on neonatal outcomes after discharge, such as neurodevelopmental results.
(2) Deepening Mechanistic Exploration and Path Analysis: Future research should utilize theoretical frameworks like the JD-R model to conduct in-depth analyses of how management resources mitigate the cognitive “bottleneck effect” in nurses to reduce clinical error risks. Efforts should be made to reveal the mediation mechanisms that link individual resilience to systemic safety.
(3) Strengthening Cost-Benefit and ROI Analysis: Nursing management decisions require not only clinical evidence but also economic justification. Future studies should attempt to quantify the contribution of interventions in reducing the sunk costs of nurse turnover (typically 1.5 to 2 times an annual salary). This provides a more intuitive decision-making reference for administrators regarding investment in human resource development.
(4) Exploring Digital and AI-Driven Empowerment Strategies: In alignment with nursing informatics trends, researchers should validate the empirical effects of Artificial Intelligence (AI) in optimizing scheduling, providing early stress warnings, and delivering psychological interventions. It is essential to explore how technological intervention can reshape the allocation of work resources in the NICU.
(5) Advancing Cross-Regional and Multi-Center Validation: Cross-national and cross-cultural comparative studies are encouraged to verify the universal applicability and cultural adaptation of existing intervention models across different healthcare systems. This will provide scientific guidance for the sustainable development of the global NICU nursing workforce.

## 5 Conclusion

This integrative review systematically evaluated management interventions targeting NICU nurse burnout, turnover intention, and patient safety. The findings confirm that the professional wellbeing of NICU nurses is not an isolated psychological issue but a critical systemic variable closely linked to neonatal clinical outcomes. Burnout is not only a core predictor driving turnover intention but also significantly associated with an increased risk of HAIs and a decline in the perceived quality of care, thereby posing a potential threat to infant safety.

Guided by the JD-R framework, effective management interventions generate powerful buffering effects through the “motivational pathway” (or gain pathway), thereby assisting in mitigating the vicious cycle of “burnout-turnover-declining safety”: (1) Organizational Level: Implementing mentoring programs and establishing clear career progression pathways, such as the Enhanced Neonatal Nurse Practitioner (ENNP) role, serve as core “job resources” to enhance job satisfaction and retention. (2) Cultural Level: Fostering a positive organizational ethical climate and a multidisciplinary collaborative environment effectively offsets the moral distress and compassion fatigue triggered by the high-intensity demands of the NICU. (3) Individual Level: Mindfulness training and meditation practices serve as necessary supplements to strengthen psychological resilience, effectively blocking the pathway from acute stress to chronic occupational burnout. Given the predominance of cross-sectional data in the current evidence base, further longitudinal research is warranted to definitively elucidate the causal trajectories among these variables.

In conclusion, nursing managers and policymakers should recognize the enhancement of nurse wellbeing as a pivotal pathway to achieving the “Quadruple Aim” in healthcare. This necessitates that while optimizing patient outcomes and controlling operational costs, the joy and meaning of clinical work must be preserved. While this review identifies powerful management resources grounded in the JD-R framework, administrators should remain cognizant of the potential for publication bias. Future research should aim to include multi-lingual and gray literature to further validate these findings across broader socio-economic landscapes. Investing in professional resources for NICU nurses remains a vital defense strategy for newborn safety and the overall quality of public health services. Future research and management practices should focus on systematically embedding these evidence-based interventions into clinical management systems to scientifically address the severe challenges posed by the global nursing workforce shortage.

## Data Availability

The original contributions presented in the study are included in the article/Supplementary material, further inquiries can be directed to the corresponding authors.
