# Correction: Management strategies to mitigate burnout and turnover intention while enhancing patient safety in neonatal intensive care: an integrative review

**DOI:** 10.3389/fpubh.2026.1892493

**Published:** 2026-06-02

**Authors:** Frontiers Production Office

**Affiliations:** Frontiers Media SA, Lausanne, Switzerland

**Keywords:** health workforce sustainability, job demands-resources model, neonatal intensive care unit, nurse burnout, occupational health, patient safety culture, public health management, turnover intention

The references were incorrectly numbered from reference 21 onwards. The numbering in the reference list has been updated, and the correct citations have been linked to the correct reference numbers in the article text.

The original version of this article has been updated.

