# Supplementary material for: Management strategies to mitigate burnout and turnover intention while enhancing patient safety in neonatal intensive care: an integrative review
Source: Front Public Health. 2026 May 25;14:1835328. doi: 10.3389/fpubh.2026.1835328 (PMC13270537; doi:10.3389/fpubh.2026.1835328)
Supplement: Supplementary file 1 [file Supplementary_file_1.docx]

**Supplementary Materials**

Content

**Table S1**  Sauerland et al. (2015)... 1

**Table S2** Noah and Potas (2022). 2

**Table S3** Clubbs et al. (2019)................................................................................. 4

**Table S4** Tawfik et al. (2017)........ 6

**Table S5** Liska et al. (2025). 7

**Table S6** Moss (2021). 9

**Table S7** Tawfik et al. (2017) 11

**Table S8** Jones and Ramsbottom (2017). 12

**Table S9** Sano et al. (2018). 13

**Table S10** Yu et al. (2021) 14

**Table S11** Trajkovski et al. (2025). 16

**Table S12** Asadollah et al. (2024). 18

**Table S13** Walden et al. (2020) 20

**Table S14** Deepak et al. (2025). 22

**Table S15** Detailed Search Strategies for Other Databases. 23

**Table S1 Sauerland et al. (2015). Assessing and Addressing Moral Distress and Ethical Climate Part II: Neonatal and Pediatric Perspectives**

| **Appraisal items (Risk of bias assessment)** | **Appraisal results** | **Score** | **Rationale and evidence** |
| --- | --- | --- | --- |
| 1. Were the criteria for inclusion in the sample clearly defined? | Yes | 1 | The participants were explicitly defined as registered nurses (RNs) working in the PICU/NICU of the academic medical center. |
| 2. Were the study subjects and the setting described in detail? | Yes | 1 | The setting was described as a 600-bed academic safety-net hospital; detailed demographic data including nurses’ age, education level, professional rank, and years of experience (seniority) were provided. |
| 3. Was the exposure measured in a valid and reliable way? | Yes | 1 | Ethical climate (exposure factor) was measured using the Hospital Ethical Climate Survey (HECS), with a Cronbach’s α of 0.94 in this study. |
| 4. Were objective, standard criteria used for measurement of the condition? | Yes | 1 | Assessment was conducted using the standardized MDS-P/N scale, which was specifically fine-tuned for pediatric and neonatal contexts. |
| 5. Were confounding factors identified? | Yes | 1 | Statistical data regarding background factors that might influence outcomes, such as education level and years of work experience, were collected and analyzed. |
| 6. Were strategies to deal with confounding factors stated? | No | 0 | The study primarily employed Pearson correlation analysis and descriptive statistics; there was no evidence of using statistical methods such as multiple regression to adjust for confounding factors. |
| 7. Were the outcomes measured in a valid and reliable way? | Yes | 1 | Moral distress (outcome measure) was measured using the MDS-P/N scale, with a Cronbach’s α of 0.93 in this study. |
| 8. Was appropriate statistical analysis used? | Yes | 1 | A Pearson correlation matrix was used to explore associations between variables, which meets the requirements for a cross-sectional correlational study. |
| Quality Grades High quality | | | |

***Note:*** This appraisal was conducted using the JBI Critical Appraisal Checklist for Analytical

Cross-Sectional Studies (2020 Version).

Quality Score = (7 / 8) × 100% = 87.5 %

Studies were classified based on the percentage of “Yes” responses: high quality (≥75%), moderate quality (50% – 74%), and low quality (< 50%).

**Table S2 Noah and Potas (2022). Association between nursing work stress, burnout and nosocomial infection rate in a neonatal intensive care unit in Hargeisa, Somaliland.**

| **Appraisal items (Risk of bias assessment)** | **Appraisal results** | **Score** | **Rationale and evidence** |
| --- | --- | --- | --- |
| 1. Were the two groups similar and recruited from the same population? | Yes | 1 | The sample consisted of 72 neonates and 45 nurses across three hospitals. Comparisons were made within the same NICU environment based on exposure levels (high vs. low burnout and stress). |
| 2. Were the exposures measured similarly to assign people to both exposed and unexposed groups? | Yes | 1 | Exposure levels for all nurses were measured using the same standardized scales (MBI and NSS). |
| 3. Was the exposure measured in a valid and reliable way? | Yes | 1 | The Maslach Burnout Inventory (MBI) and Nursing Stress Scale (NSS) were utilized, with reported Cronbach’s α coefficients of 0.80 and 0.82, respectively. |
| 4. Were confounding factors identified? | Yes | 1 | Potential confounders were identified, including nurse demographics (education, years of experience, shift work) and neonatal factors (gestational age, birth weight, and congenital malformations). |
| 5. Were strategies to deal with confounding factors stated? | Yes | 1 | Random-effects logistic regression and Poisson regression models were employed for multivariate analysis to control for confounders. Additionally, congenital disease variables were excluded in a re-analysis to eliminate major interference. |
| 6. Were the groups/participants free of the outcome at the start of the study (or at the moment of exposure)? | Yes | 1 | Exclusion criteria included neonates with maternal infections. The study focused on nosocomial infections (NI) acquired during the NICU stay, implying that the outcome was not present at the time of enrollment. |
| 7. Were the outcomes measured in a valid and reliable way? | Yes | 1 | Data on neonatal infections (bloodstream, lower respiratory tract, and urinary tract infections) were extracted directly from medical records, serving as objective clinical diagnostic indicators. |
| 8. Was the follow up time reported and sufficient to be long enough for outcomes to occur? | Yes | 1 | The five-month follow-up period was sufficient for observing the occurrence of nosocomial infections in a NICU setting. |
| 9. Was follow up complete, and if not, were the reasons to loss to follow up described and explored? | Unclear | 0 | The authors noted that data collection ceased due to the COVID-19 outbreak; however, the specific number of participants lost to follow-up or the attrition rate during the five-month period was not explicitly reported. |
| 10. Were strategies to address incomplete follow up utilized? | Unclear | 0 | Panel data analysis was utilized, but there was no detailed explanation regarding the management of bias arising from unequal follow-up durations or participant dropouts. |
| 11. Was appropriate statistical analysis used? | Yes | 1 | Random-effects panel logistic and Poisson regressions were used; a power analysis was performed, and assumptions such as normal distribution and multicollinearity were verified. |
| Quality Grades High quality | | | |

***Note:*** This appraisal was conducted using the JBI Critical Appraisal Checklist for Cohort

Studies (2017 Version).

Quality Score = (9 / 11) × 100% ≈ 81.8 %

Studies were classified based on the percentage of “Yes” responses: high quality (≥75%), moderate quality (50% – 74%), and low quality (< 50%).

**Table S3 Clubbs et al. (2019). A Community Hospital NICU Developmental Care Partner Program: Feasibility and Association With Decreased Nurse Burnout Without Increased Infant Infection Rates.**

| **Appraisal items (Risk of bias assessment)** | **Appraisal results** | **Score** | **Rationale and evidence** |
| --- | --- | --- | --- |
| 1. Is it clear in the study what is the “cause” and what is the “effect” (i.e. there is no confusion about which variable comes first)? | Yes | 1 | It was clearly established that the intervention (DCP program) preceded the measurement of outcome indicators (burnout and infection). |
| 2. Was there a control group? | No | 0 | The study employed a quasi-experimental “pre-test/post-test design,” comparing data across different time periods within the same ward without a parallel control group. |
| 3. Were participants included in any comparisons similar? | No | 0 | There was substantial turnover in the nurse population (only 34% overlap between phases); additionally, the baseline proportion of infants with NAS (Neonatal Abstinence Syndrome) differed significantly. |
| 4. Were the participants included in any comparisons receiving similar treatment/care, other than the exposure or intervention of interest? | Unclear | 0 | The authors noted that shifts in the overall NICU culture and nursing practices might have confounded the results. |
| 5. Were there multiple measurements of the outcome, both pre and post the intervention/exposure? | No | 0 | Occupational burnout (MBI-HSS) was measured only once before and once after the implementation. |
| 6. Were the outcomes of participants included in any comparisons measured in the same way? | Yes | 1 | The same MBI-HSS scale and electronic medical record (EMR) extraction methods were utilized for both the pre- and post-intervention phases. |
| 7. Were outcomes measured in a reliable way? | Yes | 1 | The MBI-HSS is a widely recognized scale, and Cronbach’s α coefficients were calculated (all were > 0.7, except for the Depersonalization (DP) dimension in the post-test). |
| 8. Was follow-up complete and if not, were differences between groups in terms of their follow-up adequately described and analyzed? | No | 0 | There was significant attrition (loss to follow-up) among the nurse participants; furthermore, independent samples analysis was used instead of paired analysis. |
| 9. Was appropriate statistical analysis used? | Yes | 1 | Statistical analyses included independent samples t-tests, Fisher’s exact tests, and effect size measures such as Relative Risk (RR). |
| Quality Grades Low quality | | | |

***Note:*** This appraisal was conducted using the JBI Critical Appraisal Checklist for Quasi-Experimental Studies (2023 Revised Version).

Quality Score = (4 / 9) × 100% = 44.4 %

Studies were classified based on the percentage of “Yes” responses: high quality (≥75%), moderate quality (50% – 74%), and low quality (< 50%).

**Table S4 Tawfik et al. (2017). Burnout in the neonatal intensive care unit and its relation to healthcare-associated infections.**

| **Appraisal items (Risk of bias assessment)** | **Appraisal results** | **Score** | **Rationale and evidence** |
| --- | --- | --- | --- |
| 1. Were the criteria for inclusion in the sample clearly defined? | Yes | 1 | Inclusion criteria were explicitly defined: staff members who had worked at a participating NICU for at least four consecutive weeks prior to the survey with a workload of≥0.5 Full-Time Equivalent (FTE). |
| 2. Were the study subjects and the setting described in detail? | Yes | 1 | The study setting (44 California NICUs) and participants (including physicians, nurses, nurse practitioners, and respiratory therapists) were described in detail; information on demographics, work shifts, and years of experience was provided. |
| 3. Was the exposure measured in a valid and reliable way? | Yes | 1 | Occupational burnout (exposure factor) was assessed using four validated emotional exhaustion items from the Maslach Burnout Inventory (MBI), a scale that has demonstrated validity in other clinical settings. |
| 4. Were objective, standard criteria used for measurement of the condition? | Yes | 1 | Standardized definitions from the CPQCC and the Vermont Oxford Network were employed to define healthcare-associated infections (HAI) in very low birth weight (VLBW) infants. |
| 5. Were confounding factors identified? | Yes | 1 | Potential confounders were identified, including infant-level clinical risk factors (sex, gestational age, Apgar scores, etc.) and provider characteristics (role, experience, work shift). |
| 6. Were strategies to deal with confounding factors stated? | Yes | 1 | Multilevel logistic regression and hierarchical modeling were utilized to adjust for infant-level clinical risk factors. |
| 7. Were the outcomes measured in a valid and reliable way? | Yes | 1 | Clinical data for HAI (outcome measure) were sourced from the CPQCC population registry, which undergoes rigorous quality checks to ensure data integrity, completeness, and accuracy. |
| 8. Was appropriate statistical analysis used? | Yes | 1 | Multilevel regression and Pearson correlation analyses were performed using SAS 9.4 to account for the nesting of provider and infant data within their respective NICUs. |
| Quality Grades High quality | | | |

***Note:*** This appraisal was conducted using the JBI Critical Appraisal Checklist for Analytical Cross-Sectional Studies (2020 Version).

Quality Score = (8 / 8) × 100% = 100 %

Studies were classified based on the percentage of “Yes” responses: high quality (≥75%), moderate quality (50% – 74%), and low quality (< 50%).

**Table S5 Liska et al. (2025). Correlation of Mindfulness Practices, Resilience, and Compassion Satisfaction in Hospital-Based Healthcare Workers: A Randomized Controlled Trial.**

| **Appraisal items (Risk of bias assessment)** | **Appraisal results** | **Score** | **Rationale and evidence** |
| --- | --- | --- | --- |
| 1. Was true randomization used for assignment of participants to treatment groups? | Yes | 1 | Manual block randomization was employed; group allocation was performed by a research assistant drawing slips of paper from opaque envelopes. |
| 2. Was allocation to treatment groups concealed? | Yes | 1 | Allocation concealment was maintained by using opaque envelopes to prevent the prior disclosure of the assignment sequence. |
| 3. Were treatment groups similar at the baseline? | Yes | 1 | Following randomization, no statistically significant differences were found between the two groups regarding demographic characteristics such as gender, age, and education level (see Table 2 in the original text). |
| 4. Were participants blind to treatment assignment? | No | 0 | As mindfulness is a behavioral intervention, participants were aware of their treatment status; therefore, blinding of participants was unfeasible. |
| 5. Were those delivering the treatment blind to treatment assignment? | Unclear | 0 | The intervention was delivered voluntarily via audio and video on a staff portal; the study does not explicitly state whether the researchers were blinded during the management of the trial. |
| 6. Were treatment groups treated identically other than the intervention of interest? | Yes | 1 | Aside from the mindfulness practice in the experimental group, both the control and experimental groups maintained their normal daily work activities. |
| 7. Were outcome assessors blind to treatment assignment? | No | 0 | Outcomes were collected via self-report scales (CD-RISC and ProQOL). Since the participants served as their own assessors and were not blinded, blinding of outcome assessors was not achieved. |
| 8. Were outcomes measured in the same way for treatment groups? | Yes | 1 | At the end of the six-week study, participants in both groups were required to complete the same set of original survey questionnaires. |
| 9. Were outcomes measured in a reliable way | Yes | 1 | Validated CD-RISC and ProQOL scales were used, and Cronbach’s α coefficients were calculated to estimate measurement reliability. |
| 10. Was follow up complete and if not, were differences between groups in terms of their follow up adequately described and analysed? | Yes | 1 | The distribution of the 14 participants lost to follow-up (all from the experimental group) and their reasons were documented in detail and presented in a CONSORT flow diagram. |
| 11. Were participants analysed in the groups to which they were randomized? | No | 0 | Seventy-three participants were randomized into the experimental group, but only the 59 who completed the study were analyzed. The 14 excluded participants were not included in the final results, indicating a per-protocol (PP) analysis. |
| 12. Was appropriate statistical analysis used? | Yes | 1 | Statistical analyses included paired t-tests, robust bootstrapping, and permutation methods; a power analysis was also reported. |
| 13. Was the trial design appropriate and any deviations from the standard RCT design (individual randomization, parallel groups) accounted for in the conduct and analysis of the trial? | Yes | 1 | The study utilized a standard parallel-group randomized controlled trial (RCT) design. |
| Quality Grades Moderate quality | | | |

***Note:*** This appraisal was conducted using the JBI Critical Appraisal Tool for Randomized Controlled Trials (2023 Revised Version).

Quality Score = (9 / 13) × 100% = 69.2 %

Studies were classified based on the percentage of “Yes” responses: high quality (≥75%), moderate quality (50% – 74%), and low quality (< 50%).

**Table S6 Moss (2021). Examining Job Satisfaction and Intent to Stay for Neonatal Nurse Practitioners: The Impact of Mentoring.**

| **Appraisal items (Risk of bias assessment)** | **Appraisal results** | **Score** | **Rationale and evidence** |
| --- | --- | --- | --- |
| 1. Is it clear in the study what is the “cause” and what is the “effect” (i.e. there is no confusion about which variable comes first)? | Yes | 1 | The study clearly identified the mentorship program as the independent variable (cause) and job satisfaction and retention intention as the dependent variables (outcomes); data were collected at multiple time points before and after the intervention. |
| 2. Was there a control group? | Yes | 1 | The study compared data between mentorship program participants (mentors and mentees) and a group of nonparticipants. |
| 3. Were participants included in any comparisons similar? | No | 0 | Participants were enrolled through voluntary application rather than random assignment; baseline data revealed significant differences in initial satisfaction scores between participants and nonparticipants, indicating self-selection bias. |
| 4.Were the participants included in any comparisons receiving similar treatment/care, other than the exposure or intervention of interest? | Yes | 1 | All participants were recruited from a single 116-bed Level IV NICU and worked within the same clinical shift environment. |
| 5.Were there multiple measurements of the outcome, both pre and post the intervention/exposure? | Yes | 1 | The study established four measurement time points: pre-implementation, six months after the conclusion of Cohort 1, at the commencement of Cohort 2, and at the conclusion of Cohort 2. |
| 6. Were the outcomes of participants included in any comparisons measured in the same way? | Yes | 1 | All groups completed their assessments using the same REDCap electronic survey platform. |
| 7. Were outcomes measured in a reliable way? | Yes | 1 | The well-established MNPJSS scale was utilized, demonstrating high internal consistency with an overall Cronbach’s α of 0.96. |
| 8. Was follow-up complete and if not, were differences between groups in terms of their follow-up adequately described and analyzed? | Yes | 1 | Valid response rates for each phase (67.5% and 53%) and fluctuations in sample size across all time points were documented in detail. |
| 9. Was appropriate statistical analysis used? | Yes | 1 | One-way repeated measures analysis of variance (ANOVA) was employed to analyze satisfaction scores, and Spearman’s rank correlation was used to explore the relationships between variables. |
| Quality Grades High quality | | | |

***Note:*** This appraisal was conducted using the JBI Critical Appraisal Checklist for Quasi-Experimental Studies (2023 Revised Version).

Quality Score = (8 / 9) × 100% = 88.9 %

Studies were classified based on the percentage of “Yes” responses: high quality (≥75%), moderate quality (50% – 74%), and low quality (< 50%).

**Table S7 Tawfik et al. (2017). Factors Associated With Provider Burnout in the NICU.**

| **Appraisal items (Risk of bias assessment)** | **Appraisal results** | **Score** | **Rationale and evidence** |
| --- | --- | --- | --- |
| 1. Were the criteria for inclusion in the sample clearly defined? | Yes | 1 | Inclusion criteria were explicitly defined: staff members working in a participating NICU for at least four consecutive weeks prior to the survey with a workload of≥0.5 Full-Time Equivalent (FTE). |
| 2. Were the study subjects and the setting described in detail? | Yes | 1 | The study provided a detailed description of the 41 California NICUs participating in the CPQCC and the 1,934 participants, which included physicians, nurses, nurse practitioners (NPs), and respiratory therapists. |
| 3. Was the exposure measured in a valid and reliable way? | Yes | 1 | Burnout was assessed using four emotional exhaustion items from the validated Maslach Burnout Inventory (MBI), yielding a Cronbach’s α of 0.85. |
| 4. Were objective, standard criteria used for measurement of the condition? | Yes | 1 | Standardized definitions from the CPQCC and the Vermont Oxford Network were used to define clinical data; burnout was operationalized as the proportion of participants with a scale score≥50. |
| 5. Were confounding factors identified? | Yes | 1 | Potential confounders were identified, including infant-level clinical risk factors (e.g., sex, gestational age, birth weight) and provider background characteristics (role, experience). |
| 6. Were strategies to deal with confounding factors stated? | Yes | 1 | Multilevel logistic regression and hierarchical modeling were applied, utilizing LASSO generalized regression models for variable selection to refine the model. |
| 7. Were the outcomes measured in a valid and reliable way? | Yes | 1 | Clinical outcome data were sourced from the CPQCC population registry and the OSHPD database, both of which undergo rigorous quality checks to ensure data accuracy and integrity. |
| 8. Was appropriate statistical analysis used? | Yes | 1 | Statistical analyses, including t-tests, correlation analysis, and multilevel regression, were conducted using SAS 9.4 and JMP Pro 13.0 software. |
| Quality Grades High quality | | | |

***Note:*** This appraisal was conducted using the JBI Critical Appraisal Checklist for Analytical Cross-Sectional Studies (2020 Version).

Quality Score = (8 / 8) × 100% = 100 %

Studies were classified based on the percentage of “Yes” responses: high quality (≥75%), moderate quality (50% – 74%), and low quality (< 50%).

**Table S8 Jones and Ramsbottom (2017). Increasing staff retention by facilitating neonatal nurse development to an enhanced level.**

| **Appraisal items (Risk of bias assessment)** | **Appraisal results** | **Score** | **Rationale and evidence** |
| --- | --- | --- | --- |
| 1. Is the source of the opinion clearly identified? | Yes | 1 | Author information is clearly stated: Tracey Jones is a Lecturer in Neonatal Nursing at the University of Manchester; Hannah Ramsbottom is an Enhanced Neonatal Nurse Practitioner (ENNP) in the Neonatal Intensive Care Unit (NICU) at St Mary’s Hospital. |
| 2. Does the source of opinion have standing in the field of expertise? | Yes | 1 | The authors hold advanced qualifications, including a Master’s degree in Nursing and Senior Fellowship of the Higher Education Academy (SFHEA); they possess extensive expertise in both academic teaching and clinical practice (ENNP). |
| 3. Are the interests of the relevant population the central focus of the opinion? | Yes | 1 | The primary focus of the article is on the professional development, retention, and empowerment of neonatal nurses, with the ultimate objective of ensuring high-quality nursing care and infant safety. |
| 4. Does the opinion demonstrate a logically defended argument to support the conclusions drawn? | Yes | 1 | The article presents a coherent logical framework: nursing shortages lead to burnout → empowerment and Continuing Professional Development (CPD) improve job satisfaction → the specific ENNP role serves as an effective pathway to achieve these goals. |
| 5. Is there reference to the extant literature? | Yes | 1 | A total of 26 references are cited, including Bliss reports, BAPM (British Association of Perinatal Medicine) standards, Office for National Statistics (ONS) data, and numerous academic nursing studies. |
| 6. Is any incongruence with the literature/sources logically defended? | Yes | 1 | The perspectives are consistent with mainstream literature (e.g., the positive correlation between CPD and retention rates); the authors provide a balanced/dialectical analysis of the distinction between the ANNP (medicalized pathway) and the ENNP (nursing-focused pathway), maintaining a robust professional stance. |
| Quality Grades High quality | | | |

***Note:*** This appraisal was conducted using the JBI Critical Appraisal Checklist for Textual Evidence and Opinion (2020/2025 Version) .

Quality Score = (6 / 6) × 100% = 100 %

Studies were classified based on the percentage of “Yes” responses: high quality (≥75%), moderate quality (50% – 74%), and low quality (< 50%).

**Table S9 Sano et al. (2018). Negative Consequences of Providing Nursing Care in the Neonatal Intensive Care Unit.**

| **Appraisal items (Risk of bias assessment)** | **Appraisal results** | **Score** | **Rationale and evidence** |
| --- | --- | --- | --- |
| 1. Were the criteria for inclusion in the sample clearly defined? | Yes | 1 | The inclusion criteria were clearly defined: Registered Nurses (RNs) providing direct patient care in Level III or IV NICUs located in the Midwest region. |
| 2. Were the study subjects and the setting described in detail? | Yes | 1 | The study background (Midwest US NICUs) and sample characteristics were described in detail, including age, gender, work experience (mean = 18.40 years), educational level (84% with a Bachelor’s degree or higher), and work intensity. |
| 3. Was the exposure measured in a valid and reliable way? | Yes | 1 | Validated scales were used to measure primary predictors: the PES-NWI Collaboration subscale (Cronbach’s α = 0.81) and the Self-Compassion Scale (α = 0.93). Although “nurse-patient relationship strength” was measured using a single item, its use was justified through cited literature. |
| 4. Were objective, standard criteria used for measurement of the condition? | Yes | 1 | While categorized as psycho-behavioral research rather than clinical diagnosis, the study utilized widely recognized standard scales (ProQOL, STSS, and OLBI) to assess negative professional outcomes among nurses. |
| 5. Were confounding factors identified? | Yes | 1 | The study identified and collected data on potential confounders that might influence results, such as age, education, years of experience, hospital Magnet status, and employment status (full-time vs. part-time). |
| 6. Were strategies to deal with confounding factors stated? | Yes | 1 | Missing data were addressed using multiple imputation; subsequently, the aforementioned background variables were incorporated as covariates in the Structural Equation Modeling (SEM) analysis. |
| 7. Were the outcomes measured in a valid and reliable way? | Yes | 1 | All three outcome indicators were measured using high-reliability scales: Compassion Fatigue (α = 0.84), Secondary Traumatic Stress (α = 0.92), and Burnout (α = 0.81). |
| 8. Was appropriate statistical analysis used? | Yes | 1 | Advanced statistical techniques, including Moderated Mediation Analysis and Structural Equation Modeling (SEM), were employed; significance was tested using 95% bootstrapping confidence intervals. |
| Quality Grades High quality | | | |

***Note:*** This appraisal was conducted using the JBI Critical Appraisal Checklist for Analytical Cross-Sectional Studies (2020 Version) .

Quality Score = (8 / 8) × 100% = 100 %

Studies were classified based on the percentage of “Yes” responses: high quality (≥75%), moderate quality (50% – 74%), and low quality (< 50%).

**Table S10 Yu et al. (2021). Predictors of nurse-reported quality of care in neonatal intensive care units in Korea.**

| **Appraisal items (Risk of bias assessment)** | **Appraisal results** | **Score** | **Rationale and evidence** |
| --- | --- | --- | --- |
| 1. Were the criteria for inclusion in the sample clearly defined? | Yes | 1 | Inclusion criteria were explicitly stated: holding a Korean Registered Nurse license, currently working in a NICU, being proficient in reading and writing Korean, and providing voluntary consent. Exclusion criteria included nurse managers, as they do not provide direct patient care. |
| 2. Were the study subjects and the setting described in detail? | Yes | 1 | The study participants were NICU nurses recruited from four tertiary hospitals and three general hospitals across four cities in South Korea. Table 1 provided detailed demographic data, including age, gender, education level, and years of nursing experience. |
| 3. Was the exposure measured in a valid and reliable way? | Yes | 1 | Perceptions of staffing were measured using the AHRQ Hospital Survey on Patient Safety Culture (HSOPS). Turnover intention and job satisfaction were assessed via single-item measures, with their predictive validity supported by existing literature cited in the text. |
| 4. Were objective, standard criteria used for measurement of the condition? | Yes | 1 | Nursing Quality of Care (NQoC) was evaluated using standardized questionnaire items widely recognized in the field, such as those derived from the RN4CAST study. |
| 5. Were confounding factors identified? | Yes | 1 | Researchers identified a strong correlation between total nursing experience and NICU-specific experience. Additionally, background factors such as education level, hospital type, and overtime work were collected as covariates for the analysis. |
| 6. Were strategies to deal with confounding factors stated? | Yes | 1 | Hierarchical logistic regression was employed to examine the relationships between variables. To prevent multicollinearity, total nursing experience was excluded from the model due to its high correlation with NICU-specific experience. |
| 7. Were the outcomes measured in a valid and reliable way? | Yes | 1 | The measurement of the outcome variable (NQoC) has been validated in cross-sectional studies across multiple countries; its validity was further substantiated through various literature citations within the study. |
| 8. Was appropriate statistical analysis used? | Yes | 1 | G-Power was used to calculate the required sample size to ensure adequate statistical power. Statistical methods included t-tests, chi-square tests, Fisher’s exact tests, and hierarchical logistic regression; Hosmer-Lemeshow goodness-of-fit indicators were also reported. |
| Quality Grades High quality | | | |

***Note:*** This appraisal was conducted using the JBI Critical Appraisal Checklist for Analytical

Cross-Sectional Studies (2020 Version) .

Quality Score = (8 / 8) × 100% = 100 %

Studies were classified based on the percentage of “Yes” responses: high quality (≥75%), moderate quality (50% – 74%), and low quality (< 50%).

**Table S11 Trajkovski et al. (2025). Strengthening the Neonatal Workforce Through World Café Methodology.**

| **Appraisal items (Risk of bias assessment)** | **Appraisal results** | **Score** | **Rationale and evidence** |
| --- | --- | --- | --- |
| 1. Is there congruity between the stated philosophical perspective and the research methodology? | Yes | 1 | The study adopted a qualitative participatory research design combined with the World Café methodology for knowledge co-construction; the philosophical stance is congruent with the chosen methodology. |
| 2. Is there congruity between the research methodology and the research question or objectives? | Yes | 1 | The research objective was to generate strategies for improving recruitment and retention; the World Café method is highly suitable for eliciting diverse perspectives and generating actionable recommendations. |
| 3. Is there congruity between the research methodology and the methods used to collect data? | Yes | 1 | A typical World Café process was followed, involving data collection through six rotational discussion groups and guided questions; data were recorded on flip charts and sticky notes. |
| 4. Is there congruity between the research methodology and the representation and analysis of data? | Yes | 1 | Inductive thematic analysis based on the Braun and Clarke framework was utilized for coding; the analysis was conducted collaboratively by experienced researchers to ensure methodological rigor. |
| 5. Is there congruity between the research methodology and the interpretation of results? | Yes | 1 | The findings were synthesized into four core themes, providing a strategic framework for nursing leaders; the interpretation of results is consistent with the logic of qualitative inquiry. |
| 6. Is there a statement locating the researcher culturally or theoretically? | Yes | 1 | The text explicitly states that the research team comprised senior neonatal nurses, midwives, academics, and clinical researchers. |
| 7. Is the influence of the researcher on the research, and vice- versa, addressed? | Yes | 1 | The research team engaged in reflexive practice and noted that facilitators did not participate in the discussions to maintain the independence of the analysis. |
| 8. Are participants, and their voices, adequately represented? | Yes | 1 | The study included numerous verbatim quotes from participants, and Figure 1 presented the raw data generated during the sessions. |
| 9. Is the research ethical according to current criteria or, for recent studies, and is there evidence of ethical approval by an appropriate body? | Yes | 1 | Ethical approval was granted by the Western Sydney University Human Research Ethics Committee (No: H15910), and written informed consent was obtained from all participants. |
| 10. Do the conclusions drawn in the research report flow from the analysis, or interpretation, of the data? | Yes | 1 | The conclusions summarized shared responsibilities across various stakeholders, and all recommendations were directly derived from and validated by the workshop findings. |
| Quality Grades High quality | | | |

***Note:*** This appraisal was conducted using the JBI Critical Appraisal Checklist for Qualitative

Research (2015/2020 Version).

Quality Score = (10 / 10) × 100% = 100 %

Studies were classified based on the percentage of “Yes” responses: high quality (≥75%), moderate quality (50% – 74%), and low quality (< 50%).

**Table S12 Asadollah et al. (2024). The Impact of Loving-Kindness Meditation on Job-Related Burnout of Nurses Working in Neonatal Intensive Care Unit: A Randomized Clinical Trial Study.**

| **Appraisal items (Risk of bias assessment)** | **Appraisal results** | **Score** | **Rationale and evidence** |
| --- | --- | --- | --- |
| 1. Was true randomization used for assignment of participants to treatment groups? | Yes | 1 | The study described using a simple lottery method: the names of eligible nurses were placed into sealed envelopes and drawn for group allocation. |
| 2. Was allocation to treatment groups concealed? | Yes | 1 | The use of “sealed envelopes” was explicitly mentioned to ensure allocation concealment. |
| 3. Were treatment groups similar at the baseline? | Yes | 1 | Table 1 indicates that there were no statistically significant differences between the two groups regarding age, years of experience, education level, or marital status (P > 0.05). |
| 4. Were participants blind to treatment assignment? | No | 0 | Given that the intervention consisted of specific meditation audio, participants were aware of whether they received meditation or health education; therefore, blinding of participants was not feasible. |
| 5. Were those delivering the treatment blind to treatment assignment? | Unclear | 0 | The researchers delivered different files via WhatsApp, and there was no mention of whether those administering the intervention were blinded. |
| 6. Were treatment groups treated identically other than the intervention of interest? | Yes | 1 | Both groups received files via the same platform (WhatsApp) with a consistent frequency of three times per week for a duration of one month. |
| 7. Were outcome assessors blind to treatment assignment? | No | 0 | Outcomes (MBI scale) were measured via online self-assessment; since the nurses served as their own assessors and were aware of their group assignments, blinding of outcome assessors was not achieved. |
| 8. Were outcomes measured in the same way for treatment groups? | Yes | 1 | Both groups completed online assessments before and after the intervention using the same Maslach Burnout Inventory (MBI). |
| 9. Were outcomes measured in a reliable way | Yes | 1 | A Persian version of the MBI with established validity and reliability was utilized, demonstrating a Cronbach’s α of 0.9 and a test-retest reliability of 0.93. |
| 10. Was follow up complete and if not, were differences between groups in terms of their follow up adequately described and analysed? | Yes | 1 | The flow diagram detailed the reasons for attrition (3 withdrawals and 1 incomplete questionnaire), resulting in a final analysis of 33 participants per group. |
| 11. Were participants analysed in the groups to which they were randomized? | Yes | 1 | The principle of Intention-to-Treat (ITT) analysis was applied; the final number of participants analyzed was consistent with the number of successfully randomized individuals. |
| 12. Was appropriate statistical analysis used? | Yes | 1 | Statistical analyses, including paired t-tests, independent t-tests, chi-square tests, and Analysis of Variance (ANOVA), were performed, and significance levels were reported. |
| 13. Was the trial design appropriate and any deviations from the standard RCT design (individual randomization, parallel groups) accounted for in the conduct and analysis of the trial? | Yes | 1 | A parallel-group randomized clinical trial design was adopted, which is appropriate for the specialized clinical environment of the NICU. |
| Quality Grades High quality | | | |

***Note:*** This appraisal was conducted using the JBI Critical Appraisal Tool for Randomized Controlled Trials (2023 Version).

Quality Score = (10 / 13) × 100% = 76.92 %

Studies were classified based on the percentage of “Yes” responses: high quality (≥75%), moderate quality (50% – 74%), and low quality (< 50%).

**Table S13 Walden et al. (2020). What Keeps Neonatal Nurses Up at Night**

**and What Gets Them Up in the Morning?**

| **Appraisal items (Risk of bias assessment)** | **Appraisal results** | **Score** | **Rationale and evidence** |
| --- | --- | --- | --- |
| 1. Is there congruity between the stated philosophical perspective and the research methodology? | Yes | 1 | The study explicitly adopted the Transactional Model of Stress and Coping (TMSC) as its conceptual framework and utilized a descriptive research methodology, demonstrating logical consistency. |
| 2. Is there congruity between the research methodology and the research question or objectives? | Yes | 1 | The research objective was to explore nurses’ professional satisfaction and concerns; a descriptive qualitative design was both congruent and appropriate for this purpose. |
| 3. Is there congruity between the research methodology and the methods used to collect data? | Yes | 1 | Subjective narratives were collected via open-ended questions within an online survey, which aligns with the data acquisition requirements of descriptive research. |
| 4. Is there congruity between the research methodology and the representation and analysis of data? | Yes | 1 | Thematic analysis was employed for data extraction and synthesis, adhering to the analytical standards of qualitative descriptive inquiry. |
| 5. Is there congruity between the research methodology and the interpretation of results? | Yes | 1 | The interpretation of findings was closely centered on the TMSC framework, and emergent themes were extensively compared with existing literature in the discussion section. |
| 6. Is there a statement locating the researcher culturally or theoretically? | No | 0 | While the authors’ professional credentials (e.g., PhD, NNP-BC) were listed, there was a lack of an explicit statement regarding the researchers’ personal values or cultural perspectives. |
| 7. Is the influence of the researcher on the research, and vice- versa, addressed? | Unclear | 0 | The “Limitations” section acknowledged that researcher bias might influence theme naming and noted that consensus was reached through group discussion; however, the reciprocal influence between the researcher and participants was not discussed in depth, indicating insufficient reflexivity. |
| 8. Are participants, and their voices, adequately represented? | No | 0 | Results were primarily presented through themes, sub-categories, and charts; however, there was a notable absence of verbatim quotes from participants to support the conclusions, which is a significant omission in qualitative appraisal. |
| 9. Is the research ethical according to current criteria or, for recent studies, and is there evidence of ethical approval by an appropriate body? | Yes | 1 | It was explicitly stated that approval was granted by the hospital’s nursing research committee and the Institutional Review Board (IRB), and that participation was entirely voluntary. |
| 10. Do the conclusions drawn in the research report flow from the analysis, or interpretation, of the data? | Yes | 1 | The conclusions (e.g., strengthening organizational strategies, enhancing resilience) were derived directly from the analysis of stressors and satisfaction reported by the nurses. |
| Quality Grades Moderate quality | | | |

***Note:*** This appraisal was conducted using the JBI Critical Appraisal Checklist for Qualitative Research (2015/2020 Version).

Quality Score = (7 / 10) × 100% = 70 %

Studies were classified based on the percentage of “Yes” responses: high quality (≥75%), moderate quality (50% – 74%), and low quality (< 50%).

**Table S14 Deepak et al. (2025). Work‑Related Stress among Healthcare Providers at Neonatal Intensive Care Unit (NICU): A Multicentric Collaborative Study from Karnataka, India**

| **Appraisal items (Risk of bias assessment)** | **Appraisal results** | **Score** | **Rationale and evidence** |
| --- | --- | --- | --- |
| 1. Were the criteria for inclusion in the sample clearly defined? | Yes | 1 | Inclusion criteria (e.g., pediatric or neonatal specialization) and exclusion criteria (e.g., exclusion of those with less than 3 months of NICU experience) were explicitly listed. |
| 2. Were the study subjects and the setting described in detail? | Yes | 1 | A detailed description was provided of participants recruited from eight tertiary hospitals in Karnataka. Table 1 presented comprehensive sociodemographic and occupational data, including age, gender, education, and specialty. |
| 3. Was the exposure measured in a valid and reliable way? | Yes | 1 | “Exposure” in this context refers to factors influencing stress. The study measured objective indicators such as monthly mortality rates, trainee status, and working hours, with the data sources clearly specified. |
| 4. Were objective, standard criteria used for measurement of the condition? | Yes | 1 | Work-related stress and psychological distress were measured using the TAWS-16 and K10 scales, respectively; the text explicitly states that these are validated standard instruments. |
| 5. Were confounding factors identified? | Yes | 1 | Variables potentially influencing outcomes, such as age, gender, stressful life events in the past year, and relaxation practices, were identified as explanatory variables. |
| 6. Were strategies to deal with confounding factors stated? | Yes | 1 | Multivariate logistic regression analysis was employed to control for confounding factors and determine independent associations. |
| 7. Were the outcomes measured in a valid and reliable way? | Yes | 1 | Outcome variables (stress and distress levels) were obtained using the validated TAWS-16 and K10 tools; research center investigators assisted in the survey process to ensure reliability. |
| 8. Was appropriate statistical analysis used? | Yes | 1 | P-values and 95% CIs were utilized. Statistical methods included Chi-square tests and Spearman correlation for associations, and logistic regression for identifying predictors; the methodology was scientifically sound and appropriate. |
| Quality Grades High quality | | | |

***Note:*** This appraisal was conducted using the JBI Critical Appraisal Checklist for Analytical Cross-Sectional Studies (2020 Version).

Quality Score = (8 / 8) × 100% = 100 %

Studies were classified based on the percentage of “Yes” responses: high quality (≥75%), moderate quality (50% – 74%), and low quality (< 50%).

**Table S15 Detailed Search Strategies for Other Databases**

| **Database** | **Search Strategy** |
| --- | --- |
| CINAHL (via EBSCO) | S1: (MH "Intensive Care Units, Neonatal") OR (MH "Neonatal Nursing") OR (MH "Nurses, Neonatal") OR TI ( NICU OR "neonatal intensive care" OR "neonatal nurs*" OR "neonatal intensive care nurs*") OR AB ( NICU OR "neonatal intensive care" OR "neonatal nurs*" OR "neonatal intensive care nurs*") |
|  | S2: (MH "Burnout, Professional") OR (MH "Personnel Turnover") OR (MH "Compassion Fatigue") OR (MH "Empathy") OR TI ( burnout OR "emotional exhaustion" OR depersonalization OR "turnover intention" OR "intention to leave" OR "personnel turnover" OR "compassion fatigue" OR "secondary traumatic stress" OR resilience OR retention ) OR AB ( burnout OR "emotional exhaustion" OR depersonalization OR "turnover intention" OR "intention to leave" OR "personnel turnover" OR "compassion fatigue" OR "secondary traumatic stress" OR resilience OR retention ) |
|  | S3: S1 AND S2 |
|  | Filters: English Language; Publication Date: 20150101-20251130 |
| Web of Science (Core Collection) | #1: TI=(NICU OR "neonatal intensive care" OR "neonatal nurs*" OR "neonatal intensive care nurs*") OR AB=(NICU OR "neonatal intensive care" OR "neonatal nurs*" OR "neonatal intensive care nurs*") |
|  | #2: TI=(burnout OR "emotional exhaustion" OR depersonalization OR "turnover intention" OR "intention to leave" OR "personnel turnover" OR "compassion fatigue" OR "secondary traumatic stress" OR resilience OR retention) OR AB=(burnout OR "emotional exhaustion" OR depersonalization OR "turnover intention" OR "intention to leave" OR "personnel turnover" OR "compassion fatigue" OR "secondary traumatic stress" OR resilience OR retention) |
|  | #3: #1 AND #2 |
|  | Filters: Language: English; Timespan: 2015-01-01 to 2025-11-30 |
| Embase | #1: 'newborn intensive care'/exp OR 'neonatal nursing'/exp OR 'neonatal nurse'/exp OR (NICU OR 'neonatal intensive care' OR 'neonatal nurs*' OR 'neonatal intensive care nurs*'):ti,ab |
|  | #2: 'professional burnout'/exp OR 'personnel turnover'/exp OR 'compassion fatigue'/exp OR 'empathy'/exp OR (burnout OR 'emotional exhaustion' OR depersonalization OR 'turnover intention' OR 'intention to leave' OR 'personnel turnover' OR 'compassion fatigue' OR 'secondary traumatic stress' OR resilience OR retention):ti,ab |
|  | #3: #1 AND #2 |
|  | Filters: [english]/lim AND [2015-2025]/py |
